# Multi-Antigen Viral-Vectored Vaccine Protects Against SARS-CoV-2 and Variants in a Lethal hACE2 Transgenic Mouse Model

**DOI:** 10.3390/vaccines13040411

**Published:** 2025-04-15

**Authors:** Shannon Stone, Amany Elsharkawy, J. D. Burleson, Mary Hauser, Arban Domi, Pratima Kumari, Zainab Nabi, Janhavi P. Natekar, Maciel Porto, Brian Backstedt, Mark Newman, Sreenivasa Rao Oruganti, Mukesh Kumar

**Affiliations:** 1 Department of Biology, Georgia State University, Atlanta, GA 30303, USA; sstone12@student.gsu.edu (S.S.); aelsharkawy2@student.gsu.edu (A.E.); znabi1@gsu.edu (Z.N.); jnatekar1@student.gsu.edu (J.P.N.); 2 GeoVax, Inc., Atlanta, GA 30080, USA; jburleson@geovax.com (J.D.B.); mhauser@geovax.com (M.H.); adomi@geovax.com (A.D.); pkumari@geovax.com (P.K.); mnewman@geovax.com (M.N.); 3 BioQual, Inc., Rockville, MD 20850, USA; mporto@bioqual.com (M.P.); bbackstedt@bioqual.com (B.B.)

**Keywords:** SARS-CoV-2, COVID-19, MVA-VLP

## Abstract

Widespread and rapidly evolving SARS-CoV-2 posed an unprecedented challenge to vaccine developers. GeoVax has designed a multiantigen SARS-CoV-2 vaccine, designated GEO-CM02 based on a Modified Vaccinia Virus (MVA) vector that expresses spike (S), membrane (M), and envelope (E) antigens. This experimental vaccine was tested in the hACE2 transgenic mouse model to assess immunogenicity and efficacy. Administration of the vaccine in a two-dose regimen elicited high levels of neutralizing antibodies and provided complete protection, effectively reducing lung, olfactory bulb, and brain viral load and reducing lung inflammation following infection with original B.1 virus and the B.1.1.529 variant. In addition, GEO-CM02 conferred 80% protection against a lethal infection with the B.1.351 variant. GEO-CM02 vaccine efficacy studies also demonstrated a complete level of vaccine-induced protection with a single dose against the original B.1 virus and B.1.1.529 variant. GEO-CM02 effectively elicited functional T-cell responses in both prime and prime–boost groups. These data indicate that vaccination with the GEO-CM02 vaccine can induce immune responses that protect against severe disease induced by SARS-CoV-2 and its variants in a highly relevant pre-clinical model.

## 1. Introduction

With unprecedented speed and scale, severe acute respiratory syndrome coronavirus 2 (SARS-CoV-2) caused a deadly global pandemic [1]. Rapid development, manufacture, and roll-out of first-generation vaccines afforded protection to vulnerable groups against the original strain of SARS-CoV-2 and early variants [2,3,4,5]. The primary immunogen of all first-generation vaccines is the SARS-CoV-2 spike (S), which elicits high titers of neutralizing antibody (NAb) that mediate significant levels of protection against infection and severe disease [6]. However, as new variants of concern (VOCs) arose, the ability of the first-generation vaccines and monoclonal antibodies to neutralize emerging strains was disrupted. It is now unclear the extent to which the current generation of vaccines will be able to provide high levels of efficacy. Evasion of NAb by emerging VOCs and waning antibody responses pose looming challenges for durable vaccine protection [7]. Recently, highly transmissible Omicron variant sublineages, including JN.1 and KP.2, which contain more than 30 amino acid substitutions in the S gene, with over half of these in the receptor binding domain (RBD) [8], have maintained the trend of viral evolution that evades vaccine-induced neutralizing antibodies [9,10].

The design of effective next-generation COVID-19 vaccines must consider the highly divergent and rapidly mutating nature of coronaviruses [11]. While a high level of viral escape from NAb exists among VOCs, the T cell epitopes in the S and other large structural proteins have remained largely conserved, suggesting that the current vaccines may not be rendered completely ineffective if conserved epitopes can be targeted effectively [12]. Recent studies have highlighted the importance of cellular immunity, which is maintained for up to 20 months after infection [13,14]. The S protein has historically exhibited a great degree of divergence compared to other coronavirus proteins; thus, inclusion of proteins that remain more highly conserved may offer greater protection against emerging VOCs.

Alternative vaccines based on modified antigen design or various platform technologies that focus on cellular immunity, and immunogenic targets beyond S may be able to establish a superior long-term immune profile better suited for a rapidly evolving RNA virus. Additionally, it was extensively shown that robust T-cell responses are significantly correlated with a lower degree of disease pathogenesis [15,16,17,18,19,20]. As such, broadening cellular immunity to multiple virus antigens is a feasible way to offer enhanced protection against VOCs [21,22].

There are large numbers of T cell epitopes recognized following SARS-CoV-2 infection beyond S, including nucleoprotein (N), membrane (M), and many nonstructural proteins. In particular, the M protein has been identified as a major T cell target [23]. Significant T cell reactivity against M has been documented in convalescent COVID-19 patients. Specificity to M accounted for 21% and 22% of the total CD4+ and CD8+ T cell response, respectively, making it second to S as the most recognized antigen by cellular responses [24]. When co-expressed, S, M, and envelope (E) support the spontaneous formation of virus-like particles (VLPs) [25]. VLPs are very effective for enveloped viruses, as they can elicit antibodies to native forms of viral envelope glycoproteins. Furthermore, VLPs have been demonstrated to be potent activators of dendritic cells (DCs), which play a critical role in priming both B and T cells [26,27].

The MVA-VLP vaccine combines the safety of a non-replicating virus vector with the enhanced immunogenicity of VLPs. Specifically, MVA is an ideal vaccine platform for induction of potent multi-antigen cellular immune responses as it preferentially targets antigen presenting cells in vivo [28,29,30]. MVA also presents antigens through the cross-presentation pathway, which is very effective for the delivery of stable proteins or particulates, such as VLPs, to induce humoral and cellular responses [31,32]. The MVA platform is known to be safe and immunogenic in humans [33,34] and to protect animal surrogates against a variety of human disease agents in preclinical testing [35,36]. Importantly, MVA is not hindered by pre-existing vector immunity like other replication-competent vectors. At moderate doses of MVA, no significant neutralizing antibody is detected. Furthermore, GeoVax confirmed that pre-existing vaccination with MVA does not hinder the induction of humoral immunity to a subsequent heterologous booster [37,38,39]. Herein, utilizing the MVA-VLP platform, we tested the efficacy of a multi-antigen vaccine expressing SARS-CoV-2 S, M, and E proteins in preclinical animal infection models.

## 2. Materials and Methods

### 2.1. Vaccine Construction and Characterization

The GEO-CM02 vaccine construction was performed as previously described [40] using SARS-CoV-2 virus S, M, and E sequences (GenBank Accession number MN908947.3). Briefly, SARS-CoV-2 genes were codon-optimized for the vaccinia virus genome [41]. The SARS-CoV-2 S, M, and E gene sequences were inserted with each gene under the control of separate pox viral promoters between two essential vaccinia genes (A5R and A6L) using the pLW73 shuttle vector as previously described [39].

To confirm the expression of S and M, DF1 cells were infected with GEO-CM02 or the parental MVA (empty vector) at a multiplicity of infection (MOI) of 0.5 FFU/cell for 48 h at 37 °C. Subsequently, cells were lysed, and the proteins were separated by 4–12% SDS-PAGE under denaturing and reducing conditions. Proteins were transferred to nitrocellulose, and the membrane was stained with anti-S (Sino Biologicals, T-62, Beijing, China) and anti-membrane (Genetex, GTX636245, Irvine, CA, USA) antibodies and visualized with the Li-Cor Odyssey imaging system. To confirm the formation of VLPs by electron microscopy, DF-1 cells were infected with GEO-CM02 for 24 h, stained with a rabbit anti-S antibody, fixed with 1% glutaraldehyde in 0.1 M phosphate buffer, and incubated in 50 mM glycine to block residual aldehyde. Following incubation in goat anti-rabbit secondary antibody conjugated to 10 nm gold particles (Electron Microscopy Sciences, Cat# 25108), silver enhancement was performed to increase the size of gold particles for subsequent viewing on a TEM (JEOL JEM-1400, Tokyo, Japan) microscope [40].

### 2.2. In Vivo Mouse Vaccination and Infection Experiments

Mouse immunogenicity and virus challenge experiments were conducted in ABSL-2 and ABL-3, respectively. The protocol was approved by the GSU IACUC (Protocol number A24003) and BIOQUAL IACUC (Protocol number 21-024P).

A schematic overview of the in vivo vaccination protocol, virus challenge, and specimen collection schedule for each group is provided in Supplementary Figure S1. For immunogenicity studies, mice were immunized intramuscularly with PBS or 10^7^ Plaque-Forming Units (PFUs) of the GEO-CM02 vaccine. The K18-ACE2 mice were vaccinated with either a single dose (prime) or with prime and booster (prime–boost) doses, 28 days apart. Mice were euthanized on day 28 (prime group) and day 55 (prime–boost group), and samples was collected. Animals were monitored twice daily for symptoms following vaccination to assess general toxicity.

For efficacy studies, K18-ACE2 mice were vaccinated and infected using intranasal administration of PBS (mock) or 10^5^ PFUs of SARS-CoV-2, as described previously, 28 days following prime and prime–boost vaccinations [42]. We used B.1 Wuhan virus (BEI# 52281), the B.1.351 variant (BEI# NR-54008), or the B.1.1.529 variant (BEI# NR-56461). Animals were observed twice daily for weight loss, breathing or appetite changes, and neurological signs. The designation for the health scores is as follows: 1, ruffled fur/hunched back; 2, slow/paresis; 3, difficulty walking/paralysis; 4, moribund/euthanized; and 5, dead. On days 3 and 6 after infection, animals were euthanized, and different tissues were collected for further analysis as described below.

### 2.3. Plaque Reduction Neutralization Test (PRNT)

Serum collected from the mice was used to quantify antibody production against SARS-CoV-2 after vaccination using the plaque reduction neutralization test (PRNT) [43]. Serum was diluted serially from 1:10 to 1:5120 using 4-fold dilutions following an initial 10-fold dilution. Diluted serum was incubated with SARS-CoV-2 virus of known plaque concentration to allow for neutralization prior to adding onto Vero 76 or Vero TMPRSS2 cell monolayers in 24-well plates for the plaque assay.

### 2.4. Binding Antibody

Total SARS-CoV-2 binding anti-S, M, and E IgG titers in sera from vaccinated mice were measured by enzyme-linked immunosorbent assay (ELISA) [40]. Total antibody binding concentration was calculated using a standard curve generated using total mouse IgG used in ELISA.

### 2.5. T Cell Analysis

Intracellular cytokine (ICS) flow assay was used to detect the S and membrane-specific cellular immune responses induced by the GEO-CM02 vaccine as described previously [39]. Briefly, mice were vaccinated with two doses, 28 days apart. Mice were euthanized on day 28 (prime group) and day 55 (prime–boost group), and single-cell suspension of spleens were prepared by homogenizing spleens and passing them through 70 µm cell strainers; after RBC lysis, 10^6^ splenocytes were stimulated with SARS-CoV-2 B.1 S or membrane peptide pools (1 μg/mL) (JPT Peptide Technologies, Berlin, Germany). After 2 h at 37 °C, brefeldin A and monensin were added (10 μg/mL), and incubation was continued for 6 h. Cells were stained with Live/Dead FITC dye (ThermoFisher; L23101), CD3, CD4, CD8, IFN-γ, IL-2, and IL4 antibodies, each conjugated to a different fluorochrome for 30 min at 4 °C. CD44 and CD62L antibodies were used to identify the naïve (CD44^low^, CD62L^high^), effector (CD44^high^, CD62L^low^), and central (CD44^high^, CD62L^high^) memory phenotype in unvaccinated and vaccinated mouse splenocytes. Approximately 100,000–200,000 lymphocytes were acquired on the Attune NxT flow cytometer and analyzed for S or membrane-specific CD4 and CD8 T cells using FlowJo^TM^ 10 software.

### 2.6. Viral Load Analysis

The lungs and brain tissues were harvested from SARS-CoV-2 infected animals and flash-frozen, following cardiac profusion with 1X PBS. The tissues were weighed and homogenized in a Bead Mill Homogenizer (Fisherbrand, Cat# 15-340-163, Waltham, MA, USA). Virus titers were measured by plaque assay using Vero E6 cells [42,44]. RNA was isolated from various tissues using a Qiagen RNA extraction kit. To determine the expression levels of the SARS-CoV-2 N1 gene, RT-qPCR was used [45]. The quantity of viral N1 gene copies was determined by comparing them to a standard curve [46].

### 2.7. Immunohistochemistry

Lung and brain tissues were harvested and fixed in 4% Paraformaldehyde (PFA) for 24 h. Tissues were then embedded in OCT medium and stored in −80 °C. Then, 5 μm sagittal sections were cut from the brain or from the left lung collected from infected mice. Lung tissue sections were stained with hematoxylin and eosin (H&E) (Abcam, Cat# ab245880, Waltham, MA, USA) and anti-SARS-CoV-2 N protein antibody (Cell signaling Technology, Cat# 26369 (HL344), Danvers, MA, USA), followed by incubation with Goat anti-Rabbit IgG (H + L) Cross-Adsorbed Secondary Antibody, Alexa Fluor™ 555, for 1 h at RT (Thermo Fisher Scientific, Cat# A-21428, RRID AB_2535849, Waltham, MA, USA). Additionally, lung tissue sections were incubated with CD45-Alexa Fluor^®^ 488 (cell signaling technology, Cat# D3F8Q, Waltham, MA, USA) and Anti-Actin α-Smooth Muscle-Cy3™ antibody (Sigma, Cat# C6198, St. Louis, MO, USA) overnight at 4 °C. Stained sections were mounted with antifade mounting medium with DAPI. Images were acquired with the Invitrogen^TM^- EVOS^TM^ M5000 Cell Imaging System (Thermo Fisher Scientific, Waltham, MA, USA) [44].

### 2.8. Luminex

Levels of proinflammatory cytokines and chemokines in the lung tissue of mock-infected, saline-infected, and vaccine-infected mice were measured by a multiplex immunoassay using the MILLIPLEX MAP Mouse Cytokine/Chemokine kit (Millipore, St. Louis, MO, USA) [44,47,48].

### 2.9. Statistical Analysis

GraphPad Prism 10 was used to perform a Kaplan–Meier log-rank test to compare survival curves. Body weight change *p* values were calculated using two-way analysis of variance (ANOVA) with the post hoc Bonferroni test. Differences between antibody binding, antibody neutralization, viral titers, and immune responses were determined using the Mann–Whitney U test and unpaired Student *t*-test.

## 3. Results

### 3.1. Design and Characterization of Multi-Antigen MVA-VLP Vaccine Candidate

The MVA-based viral vector vaccine, GEO-CM02, was developed to target SARS-CoV-2 by incorporating the S protein with the M and E viral proteins. The co-expression of these three structural proteins has been shown to result in the formation of VLPs, validating GeoVax’s MVA-VLP vaccine platform technology. A schematic representation of the viral vaccine constructs is provided in the Supplementary Materials (Figure S2A). The recombinant viral vaccine was produced under aseptic conditions using specific-pathogen-free chicken embryonic fibroblasts. Transgenes were inserted into the MVA genome via homologous recombination, with expression driven by the modified H5 (PmH5) MVA promoter. GEO-CM02 encodes a prefusion-stabilized S protein, achieved by the substitution of two proline residues at positions 986 and 987, in addition to the M and E proteins. Representative images of VLP formation are shown in Supplementary Figure S2B. DF1 cells infected with the viral vaccine were fixed, embedded, and visualized using thin-layer electron microscopy. The VLPs specifically stained for S, as indicated by positive immunogold labeling. The recombinant was characterized for the expression of S and M proteins using Western blotting (Supplementary Figure S2C,D). However, detection of the E protein was not feasible due to the unavailability of specific reagents necessary for its detection. However, the formation of VLPs suggest potential E protein production.

### 3.2. GEO-CM02 Vaccination Elicits Neutralizing Antibodies and Functional T Cell Responses in hACE2 Mice

**Figure 1:**
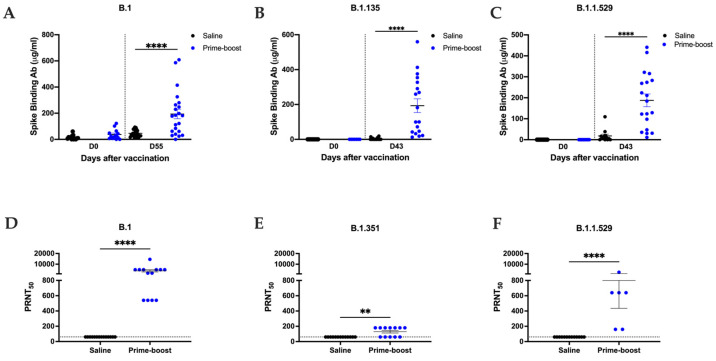
Humoral response after GEO-CM02 vaccination in hACE2 mice. (**A**–**C**) IgG binding antibody titers. Binding Ab titers against B.1-, B.1351-, or B.1.1.529-specific S proteins were measured in serum samples of vaccine and saline groups at day 0 (*n* = 14) and at days 43 or 55 (*n* = 24) after vaccination using ELISA. Data are presented as mean values ± SEM. Each point represents an individual mouse. One-way ANOVA test and Bonferroni’s test were used for determining statistical significance. (**D**–**F**). Variant-specific NAb titers. NAb titers in vaccine and saline serum collected at day 55 after vaccination were measured against B.1 (*n* = 14), B.1351 (*n* = 12), or B.1.1.529 (saline *n* = 14, prime–boost *n* = 6) live virus by PRNT assay. Titers are expressed as PRNT50. Data are presented as mean values ± SEM. Each point represents an individual mouse. Statistical significance was determined by Mann–Whitney U test (** *p* < 0.01, **** *p* < 0.0001).

The transgenic hACE2 mice were vaccinated with the GEO-CM02 vaccine at days 0 and 28. Four weeks after the booster, binding antibody responses to the full-length S proteins of the B.1, B.1.351, and B.1.1.529 variants were assessed using ELISA (Figure 1A–C). Sera from GEO-CM02-vaccinated mice on day 55 showed a robust total immunoglobulin G (IgG) binding antibody response to the B.1 S protein, with similar binding levels to the B.1.351 and B.1.1.529 variants. This finding is particularly interesting; most mutations in these variants occurred in the RBD of the S protein. Despite this, GEO-CM02-vaccinated mice maintained high levels of S-binding antibodies, suggesting that the immune response may also be targeting other parts of the S protein excluding the RBD region. However, we were not able to detect binding antibodies specific to the M and E proteins (Supplementary Figure S3).

We next assessed the neutralizing capacity of sera collected following prime–boost vaccination (day 55) of mice using live SARS-CoV-2 virus-neutralizing PRNT assay, measuring 50% reduction in plaque formation (Figure 1D–F). Sera from GEO-CM02-vaccinated mice demonstrated strong neutralizing activity towards B.1 virus (Figure 1D). However, neutralization capacity against the B.1.351 and B.1.1.529 variants reduced drastically, despite the similar binding antibody responses to the S protein across all variants. We also evaluated the neutralization activity of sera collected 28 days after the primary vaccine dose. No significant levels of neutralizing antibodies against the B.1 variant were detected in sera from mice that received only a single dose (Supplementary Figure S4).

**Figure 2:**
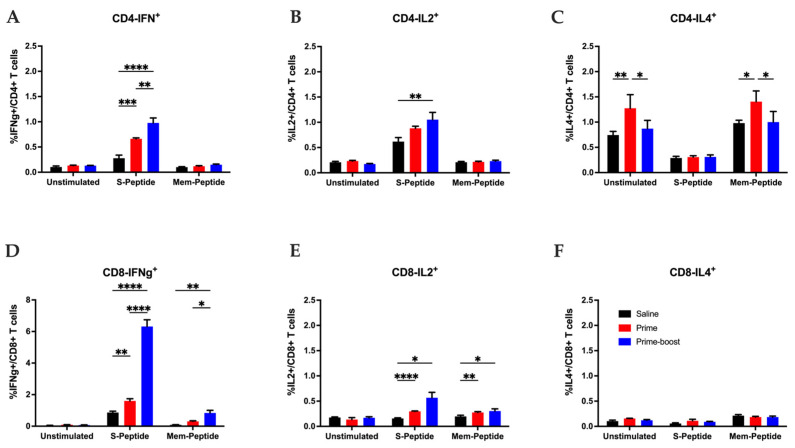
Functional SARS-CoV-2-specific T cell response after GEO-CM02 vaccination. Splenocytes were isolated from spleens collected 4 weeks following saline or GEO-CM02 prime or prime–boost vaccination (day 55) of hACE2 mice. Cells were stimulated ex vivo with B.1-specific S or M peptide pools, fixed, stained for cell surface markers (CD3, CD4, and CD8) and intracellular cytokines (IFNγ, IL-2, and IL-4) and analyzed by flow cytometry. (**A**) Percentage of CD4+ IFNγ^+^ T cells. (**B**) Percentage of CD4+ IL2+ T cells. **(C**) Percentage of CD4+ IL4^+^ T cells. (**D**) Percentage of CD8+ IFNγ^+^ T cells. (**E**) Percentage of CD8+ IL2+ T cells. (**F**) Percentage of CD8+ IL4+ T cells. Each point represents an individual mouse. The middle horizontal bar indicates the mean, and error bars are ± SEM (*n* = 4). Statistical significance was determined by two-way ANOVA, followed by Tukey’s multiple comparisons test. (* *p* < 0.05; ** *p* < 0.01; *** *p* < 0.001, **** *p* < 0.0001).

T cell functionality was evaluated by ICS using splenocytes collected 28 days following saline or GEO-CM02 prime or prime–boost vaccination (day 55). Cells were left unstimulated or stimulated ex vivo with overlapping peptide pools peptides specific to the B.1 SARS-CoV-2 S and M proteins. Gating strategy is shown in Supplementary Figure S5. The E protein was excluded from this analysis [24]. Compared to the saline control group, splenocytes from GEO-CM02-vaccinated mice exhibited a significant increase in IFN-γ-producing spike and membrane-specific CD4+ and CD8+ T cells (Figure 2A,D). The vaccination also induced an increase in IL-2-producing CD4+ and CD8+ T cells specific for the S protein (Figure 2B,E). In contrast, little to no difference was observed between saline and vaccinated groups in CD4+ and CD8+ T cells producing IL-4 following peptide stimulation (Figure 2C,F). These results suggest that GEO-CM02 vaccination generates functional CD4+ and CD8+ T cells while favoring a Th-1 immune response (as indicated by the ratio of IFN-γ+ to IL-4+ cells) over a Th-2 response.

Further analysis of central and effector memory T cell subsets was conducted on unstimulated splenocytes (Supplementary Figure S6). Naïve cells were identified as CD44^low^, CD62L^high^ central memory as CD44^high^, CD62L^high^ and effector memory as CD44^high^, and CD62L^low^. GEO-CM02 vaccination led to an increase in effector memory CD4+ and CD8+ T cells after the prime dose, with further increases observed following the booster dose. This indicates the presence of a memory T cell population and suggests the induction of long-term cellular immune responses.

### 3.3. The GEO-CM02 Vaccine Provides Protection Against Lethal SARS-CoV-2 Variants in K18-hACE2 Mice

**Figure 3:**
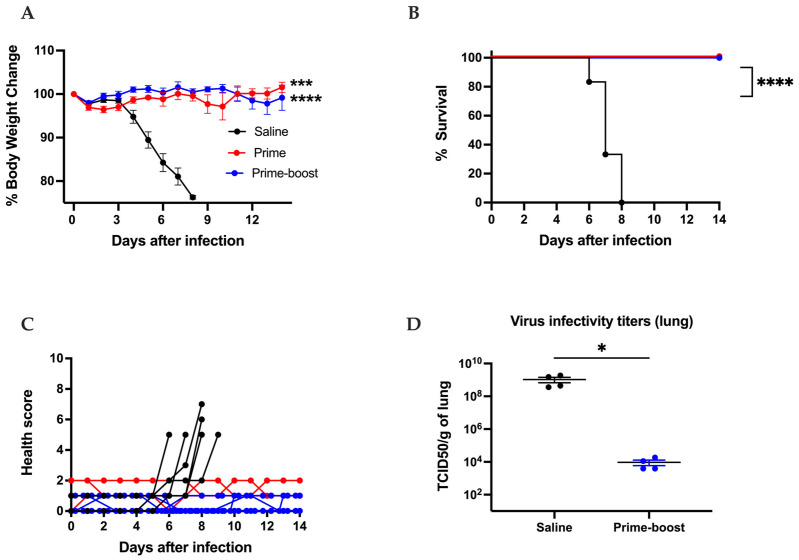
GEO-CM02 vaccine efficacy in a lethal hACE2 mouse model. Mice were vaccinated with one vaccine dose (prime) or two vaccine doses 28 days apart (prime–boost). Control group mice were vaccinated with saline. Vaccinated mice were infected with B.1 on 28 days post-prime or 56 days post-prime–boost vaccination and monitored daily for 2 weeks. (**A**) Percentage of body weight change. Ordinary one-way ANOVA followed by Dunnett’s multiple comparisons test (saline and prime–boost: *n* = 14; prime: *n* = 5) were used to determine statistical significance. (**B**) Survival. Kaplan–Meier survival curve for B.1-infected hACE-2 mice. Log-rank (Mantel–Cox) test (saline and prime–boost: *n* = 14; prime: *n* = 5) was used to determine the statistical significance. (**C**) Health scores were recorded daily following infection. (**D**) Viral titers were determined by TCID50 assay (TCID50/gram) in infected lungs collected 3 days after infection. Data are expressed as TCID50/g of tissue. The middle horizontal bar indicates the mean, and error bars are ± SEM. To determine statistical significance, an unpaired *t* test was used (*n* = 4 for each group) * *p* < 0.05, *** *p* < 0.001, **** *p* < 0.0001.

The GEO-CM02 vaccine efficacy was evaluated by utilizing a well-established lethal SARS-CoV-2 infection model using K18-hACE2 mice. A schematic presentation of the conducted studies is shown in Supplementary Figure S1. Two groups of 14 mice were vaccinated intramuscularly with either saline or 10^7^ PFUs of GEO-CM02 following a prime or prime–boost regimen on days 0 and 28. Due to the lack of sufficient vaccinated animals, a smaller GEO-CM02 prime-only (*n* = 5) group was evaluated for survival and immunogenicity but not viral loads. Animals were infected intranasally with 10^5^ PFUs of the original SARS-CoV-2 B.1 (USA_WA1/2020) on 28 days post-prime or 56 days post-prime–boost vaccination. Lungs were harvested from four euthanized animals in saline and prime–boost groups 3 days after infection for the evaluation of viral loads. Animals receiving saline exhibited significant weight loss by day 5 after infection and succumbed to disease between days 6 and 8 after infection. In contrast, both GEO-CM02 prime-only- and prime–boost-vaccinated animals were fully protected from weight loss and death (Figure 3A,B). Animals receiving prime–boost GEO-CM02 exhibited no clinical disease, whereas health scores of saline animals ranged from 5 to 7 prior to death (Figure 3D). Minimal clinical disease was observed in the prime dose group. Evaluation of lung homogenates for live SARS-CoV-2 demonstrated that saline-vaccinated animals had very high viral loads reaching 10^9^ TCID_50_/g, whereas viral loads in GEO-CM02 prime–boost-vaccinated animals remained at or near the limit of detection (Figure 3C).

**Figure 4:**
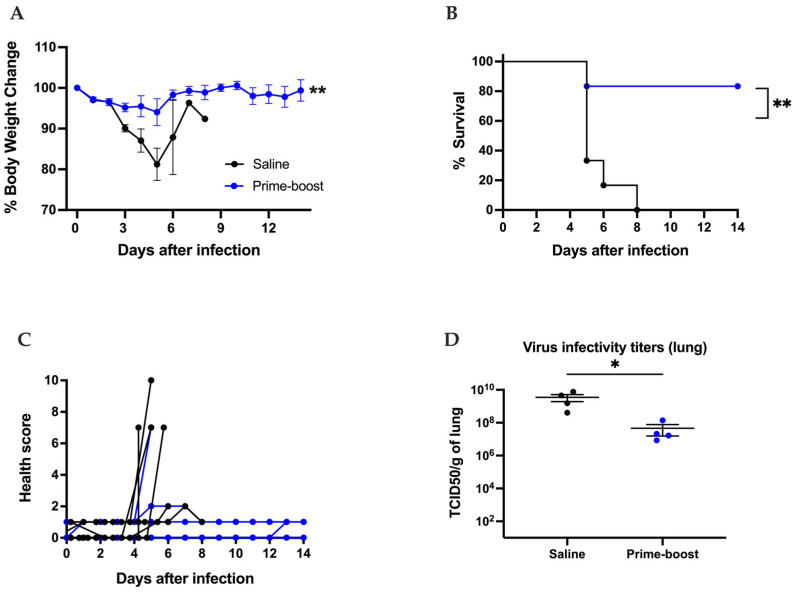
GEO-CM02 vaccine efficacy against the B.1.351 SARS-CoV-2 variant. Mice were vaccinated with two vaccine doses 28 days apart (prime–boost) or given saline as control. Vaccinated mice were infected with B.1.351 on 28 days post-prime or 56 days post-prime–boost vaccination. After infection, mice were monitored daily for 2 weeks. (**A**) Percentage of body weight change. Statistical significance was determined by Mann–Whitney U test (*n* = 12–13). (**B**) Survival. Kaplan–Meier survival curve for B.1.351-infected hACE-2 mice. Statistical significance was determined by Log-rank (Mantel–Cox) test (*n* = 12–13). (**C**) Health scores were recorded daily following infection (**D**) Viral titers were determined by TCID50 assay in infected lungs collected 3 days after infection. Data are expressed as TCID50/g of tissue. Each point represents an individual mouse. The middle horizontal bar indicates the mean, and error bars are ± SEM. Mann–Whitney U test was used to determine statistical significance (*n* = 4 for each group). * *p* < 0.05, ** *p* < 0.01.

We also evaluated the efficacy of the GEO-CM02 vaccine against the SARS-CoV-2 B.1.351 variant, which has been the most lethal variant to date [49]. Twelve to thirteen mice per group were vaccinated with saline or prime–boost of GEO-CM02 on days 0 and 28, then infected intranasally with 10^5^ PFUs of the B.1.351 variant of SARS-CoV-2 on 28 days post-prime or 56 days post-prime–boost vaccination. An additional four animals per group were vaccinated, infected, and euthanized 3 days after infection for the assessment of viral loads. B.1.351 infection was highly lethal in control hACE2 mice, with all the animals dying between days 5 and 8 after infection (Figure 4A,B). However, eighty percent of vaccinated animals survived B.1.351 lethal infection. Likewise, GEO-CM02-vaccinated animals exhibited minimal clinical disease after infection (Figure 4C). On day 3 after infection, the live virus in the lungs of vaccinated animals was significantly lower compared to the saline-vaccinated group (Figure 4D).

**Figure 5:**
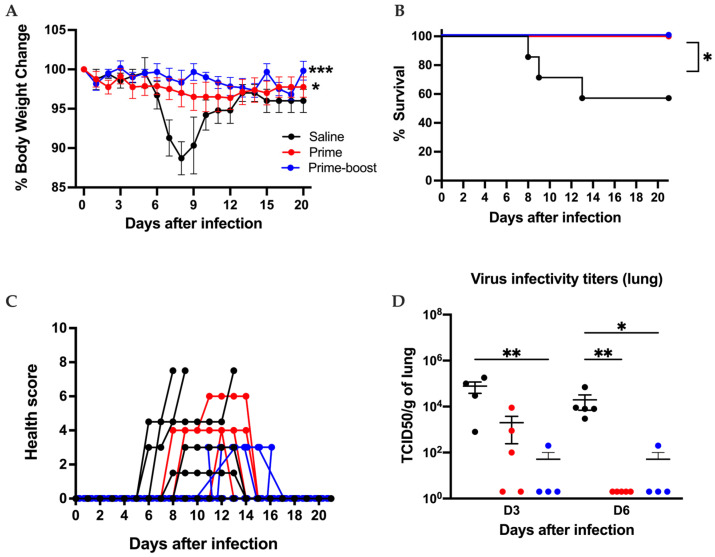
GEO-CM02 vaccine efficacy against the B.1.1.529 SARS-CoV-2 variant. Mice were vaccinated with one vaccine dose (prime) or two vaccine doses 28 days apart (prime–boost) or given saline as control. Vaccinated mice were infected with B.1.1.529 on day 28 following prime and day 56 following the prime–boost vaccinations. Following infection, mice were monitored daily for 20 days. (**A**) Percentage of body weight change. Statistical significance was determined by ordinary one-way ANOVA followed by Dunnett’s multiple comparisons test (*n* = 6–8) (**B**) Survival. Kaplan–Meier survival curve for B.1.1.529-infected hACE-2 mice. Statistical significance was determined by Log-rank (Mantel–Cox) test (*n* = 6–8). (**C**) Health scores were recorded daily following infection. (**D**) Viral titers were determined by plaque assay in infected lungs collected days 3 and 6 following infection. Data are expressed as PFU/g of tissue. Each point represents an individual mouse. The middle horizontal bar indicates the mean, and error bars are ± SEM. Kruskal–Wallis followed by Dunn’s test were used to determine statistical significance (*n* = 4–5, * *p* < 0.05, ** *p* < 0.01, *** *p* < 0.001).

Efficacy against the SARS-CoV-2 B.1.1.529 variant was also investigated by infecting 6–8 mice with 10^5^ PFUs on day 28 following prime and day 56 following the prime–boost vaccinations. An additional 8–10 mice per group were vaccinated and infected for viral titration and viral antigen detection. On days 3 and 6 after infection, 4–5 mice per group were euthanized and assessed for viral loads. Vaccinated mice remained healthy with slight weight loss at day 1 after infection, with recovery, as opposed to the saline only mice, which exhibited significant weight loss starting at day 6 after infection (Figure 5A). Four animals in the prime–boost- and seven of the prime-only-vaccinated animals displayed some clinical disease (Figure 5C) with 100% recovery and survival (Figure 5B). In comparison, significant clinical disease was observed in all the animals in the saline group.

**Figure 6:**
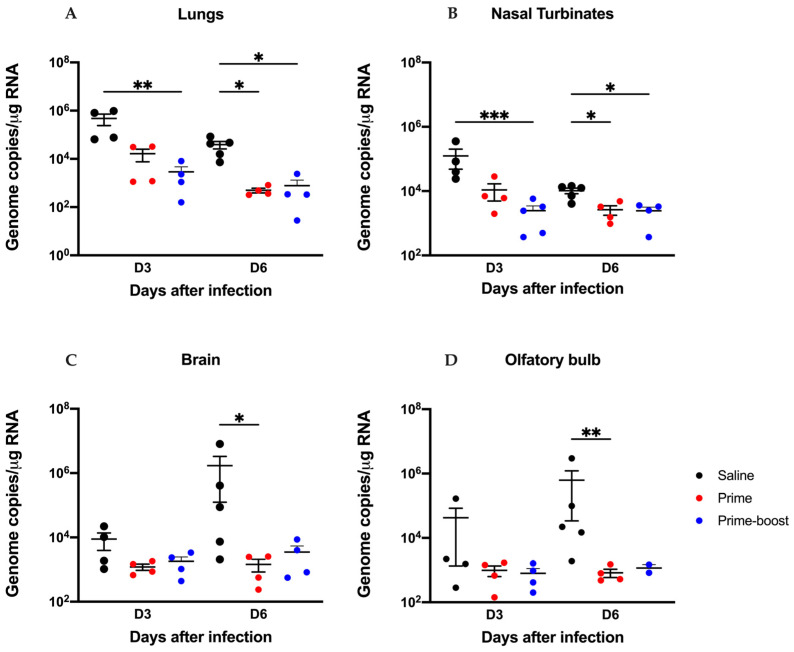
Viral titers of GEO-CM02-vaccinated and B.1.1.529 SARS-CoV-2-variant-infected mice. Mice were vaccinated with one vaccine dose (prime) or two vaccine doses 28 days apart (prime–boost) or given saline as control. Vaccinated mice were infected with B.1.1.529. Viral load was determined in (**A**) the lungs, (**B**) the nasal turbinates, (**C**) the brain, and (**D**) the olfactory bulb by RT-qPCR collected at 3 and 6 days after infection. The data are expressed on the log scale of the genomic copies/μg of RNA. The middle horizontal bar indicates the mean, and error bars are ± SEM. Statistical significance was determined by the Kruskal–Wallis test followed by Dunn’s test. * *p* < 0.05, ** *p* < 0.01, *** *p* < 0.001.

Infection with the B.1.1.529 virus resulted in 45% mortality in saline-vaccinated mice (Figure 5B). As expected, viral loads in the lung tissue of saline group were significantly higher compared to both prime and prime–boost-vaccinated animals at days 3 and 6 after infection (Figure 5D). RT-PCR was used to quantify the viral N1 gene in various tissues from these animals including lungs, nasal turbinates, brain, and olfactory bulb at days 3 and 6 after infection (Figure 6A–D). Both prime- and prime–boost-vaccinated animals exhibited a decrease in viral RNA levels at days 3 and 6 post infection in the lungs (Figure 6A), nasal turbinates (Figure 6B), brain (Figure 6C), and olfactory bulb (Figure 6D).

These results demonstrate the efficacy of the GEO-CM02 vaccine to protect hACE2 mice from both homologous and heterologous lethal SARS-CoV-2 infections. A very important finding in these studies was the complete protection from death and a reduction in viral titers with a single dose of GEO-CM02.

### 3.4. Dramatic Reduction in Lung Pathology and Inflammatory Markers in GEO-CM02-Vaccinated Mice

**Figure 7:**
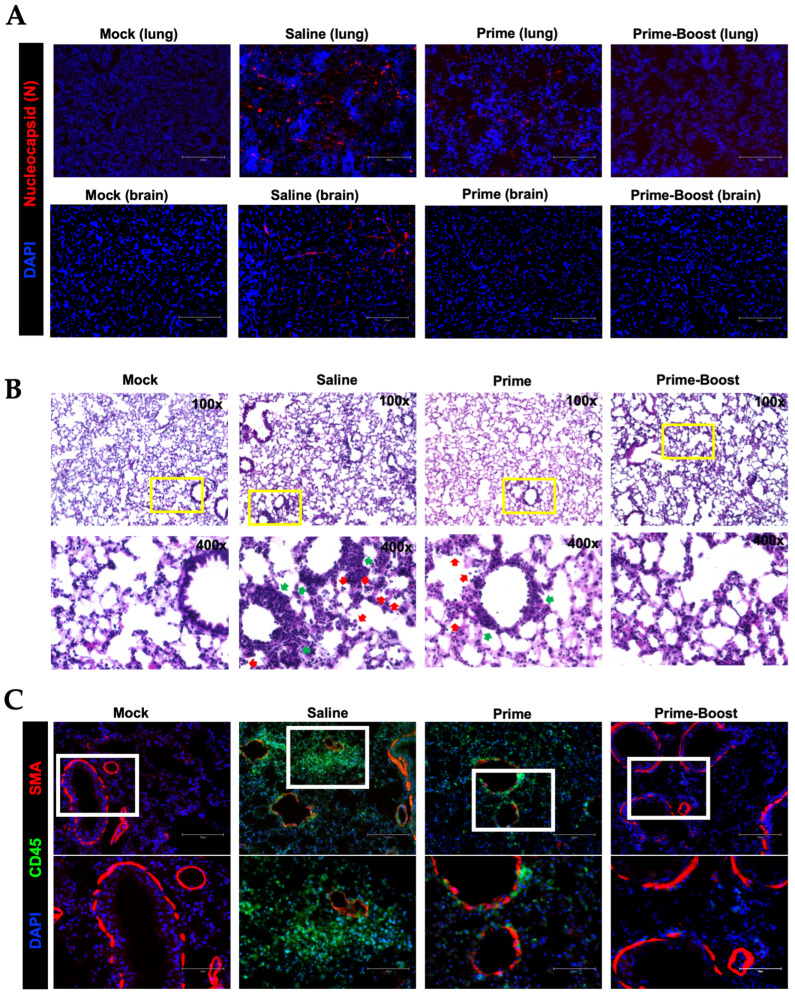
Immunopathology following SARS-CoV-2 B.1.1.529 infection in K18-hACE2 mice. Mice were vaccinated with one vaccine dose (prime) or two vaccine doses 28 days apart (prime–boost) or given saline. Vaccinated mice were infected with B.1.1.529. (**A**) Lungs were collected at day 3 after infection, and brains were collected at day 6 after infection. Infected tissues were labeled N protein (red) and DAPI (blue). Representative images are shown for each group. Scale bar is 150 μm. (**B**) Lung samples were stained with H&E. Vascular thickening (green arrows) and immune infiltrates into alveolar spaces (red arrows) are shown. Representative images are shown for each group. (**C**) Lung sections were stained with DAPI (blue), Anti-Actin α-Smooth Muscle-Cy3™ (red), and CD45-Alexa Fluor^®^ 488 (green). Scale bars: 150 μm and 75 μm on the original and high-magnification images, respectively. Representative images are shown for each group.

Analysis of infected lung and brain tissue showed an abundance of SARS-CoV-2 nucleocapsid protein distribution in the saline animals compared to the prime- and prime–boost-vaccinated animals (Figure 7A). Lung injury was also evaluated by performing H&E staining of the lung tissue collected at day 3 after infection (Figure 7B). Histopathology revealed severe virus-induced pathology, characterized by immune cell infiltration and alveolar space consolidation in the saline mice. The prime-vaccinated mice exhibited some infiltration and consolidation, while the prime–boost-vaccinated animals had no detectable immune cell infiltration or any other pathology (Figure 7B). Furthermore, analysis of the CD45 antigen revealed abundant leukocytes in the lungs of saline animals. Consistent with the H&E staining, very few CD45-positive cells were detected in the vaccinated animals (Figure 7C).

**Figure 8:**
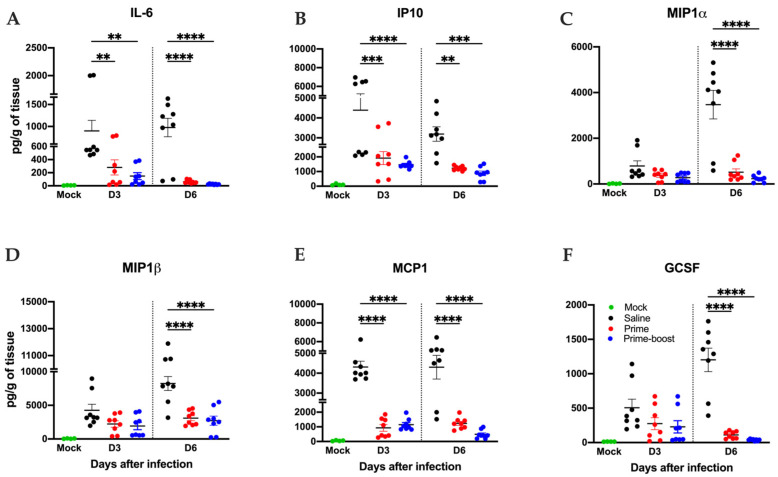
Lung inflammation following SARS-CoV-2 B.1.1.529 infection in hACE2 mice. Mice were vaccinated with one vaccine dose (prime) or two vaccine doses 28 days apart (prime–boost) or given saline. Lung homogenates were analyzed for cytokines and chemokines by Luminex assay using antibody-conjugated beads specific to the indicated proteins. Selected immune markers are shown, (**A**) IL-6, (**B**) IP10, (**C**) MIP1α, (**D**) MIP1β, (**E**) MCP1, and (**F**) GCSF. Results were quantified in pg/g of tissue. Statistical significance was determined by one-way ANOVA test, followed by Bonferroni multiple comparisons test. ** *p* < 0.01, *** *p* < 0.001, **** *p* < 0.0001.

Finally, the induction of proinflammatory cytokines and chemokines following SARS-CoV-2 infection in the lung tissues of saline and vaccinated groups was evaluated using a multiplex immunoassay (Figure 8). Compared to saline-vaccinated animals, a significant reduction in the protein levels of key proinflammatory cytokines and chemokines was observed in the prime- and prime–boost-vaccinated animals. We detected a significant decrease in the levels of IL-6, IP10, MCP1, MIPα, MIPβ, and GCSF in the prime- and prime–boost-vaccinated animals.

## 4. Discussion

The development of SARS-CoV-2 vaccines presented unique challenges due to the virus’s rapid evolution, which spurred new variants capable of evading neutralizing antibodies generated by first-generation vaccines. These early vaccines, primarily focused on the virus’s S protein, were effective against the initial strain and provided significant protection [2,3,4,5]. However, as new variants with mutations arose, they reduced vaccine effectiveness by partially evading these neutralizing antibodies. The GEO-CM02 vaccine was designed to partially address this limitation through the expanded induction of T cell responses to other structural proteins. GEO-CM02 is a multi-antigen MVA-vectored SARS-CoV-2 vaccine that targets three viral structural proteins: S, M and E. The vaccine is designed to induce both humoral and cellular responses by targeting more conserved viral regions less prone to mutation and is therefore more effective against emerging variants. The study presented here assesses the efficacy of GEO-CM02 using the hACE2 transgenic mouse model, which allows for detailed analysis of immune responses and assessment of protection against SARS-CoV-2 viral infection and its variants.

One of the critical findings that guided the development of multi-antigen vaccines was that T-cell responses remained relatively conserved across variants, even as neutralizing antibodies showed reduced efficacy. T-cell responses to SARS-CoV-2, notably to S, M, and N proteins, are robust, and highly conserved T-cell epitopes are present [50,51,52]. Studies indicate that T-cells play a critical role in protection, especially against VOCs, suggesting the potential benefit of vaccines that induce broad T-cell responses. The MVA platform is ideal for developing next-generation vaccines, as it can encode multiple immunogenic proteins and targets antigen-presenting cells effectively, promoting both CD4+ and CD8+ T-cell responses.

The protective potential of the SARS-CoV-2 E and M proteins as vaccine targets has been recently evaluated. Immunization with bovine–human parainfluenza virus type 3 expressing the S protein conferred protection against SARS-CoV, which was further enhanced by co-expression of the M and E proteins [53]. Similarly, synthetic DNA vaccines expressing either E or M provided modest protection, while co-immunization with both E and M significantly improved protection against SARS-CoV-2 infection in mice [54]. Although the SARS-CoV-2 E protein exhibits limited capacity to induce a humoral immune response, it plays a critical role in virion formation and assembly. Imaging studies have shown that co-expression of E or M proteins leads to the relocalization of the S protein to the Golgi apparatus. The contribution of E and M proteins in modulating S protein properties and facilitating VLP assembly has been previously reported [55]. More recently, specific motifs within the E protein were found to be essential for SARS-CoV-2 particle formation, further confirming its role in VLP release [56]. Consistent with these findings, our GEO-CM02 vaccine, which encodes the S, M, and E proteins, demonstrated successful formation of VLPs.

The GEO-CM02 vaccine was highly effective at inducing immune responses against SARS-CoV-2, with the following notable results: GEO-CM02 generated strong binding antibody responses against S proteins from various SARS-CoV-2 strains, including B.1, B.1.351, and B.1.1.529. On the other hand, we did not detect M- or E-specific antibodies, suggesting a limited contribution of these proteins to humoral immunity. These findings are consistent with previous reports showing minimal induction of IgG or neutralizing antibodies against SARS-CoV-2 E and M proteins in mice [53,54]. Neutralizing antibody activity against B.1.351 and B.1.1.529 was lower than that against the original B.1 virus, suggesting that while antibody responses are present, they may not be the primary driver of protection. Moreover, neutralizing antibodies against the B.1 variant were not detected in sera from mice that received only a single dose. GEO-CM02 effectively elicited functional T-cell responses in both prime and prime–boost groups, as demonstrated by an increase in IFN-γ-producing CD4+ and CD8+ T-cells. These T-cells responded to both S and M protein peptides, suggesting that the vaccine generates a broad cellular response targeting multiple viral components. Additionally, the presence of IL-2-producing T-cells highlighted the vaccine’s potential to promote a lasting immune response. Analysis of T-cell memory subsets showed a significant increase in effector memory CD4+ and CD8+ T cells after vaccination. This indicates that GEO-CM02 not only provides immediate protection but also induces a long-term immune response, which is crucial for lasting protection against reinfection. In infection studies with hACE2 mice, GEO-CM02 provided complete protection against the original B.1 virus and B.1.1.529 variant. The vaccines also provided 80% protection against the B.1.351 variant, known for its increased lethality. GEO-CM02 vaccination effectively controlled the lung and brain viral burden and reduced inflammatory cytokines in the lungs. A single dose of GEO-CM02 conferred protection without detectable neutralizing antibodies, suggesting that cellular immunity plays a significant role in vaccine efficacy.

Furthermore, the findings from GEO-CM02 studies highlight the importance of including antigens that are conserved across SARS-CoV-2 variants, such as the M protein. This strategy may help future vaccines retain efficacy as the virus continues to evolve. Additionally, the success of the MVA-VLP platform used in GEO-CM02 supports the continued exploration of VLP-based vaccines, which may be effective not only for SARS-CoV-2 but also for other rapidly mutating viruses. Viral vectors inherently activate innate immune pathways and are generally more effective at inducing robust and durable immune responses, often without the need for adjuvants. Moreover, unlike adenoviral vectors, MVA-based vaccination does not raise concerns related to vector immunity in the context of repeated dosing.

## 5. Conclusions

GeoVax’s GEO-CM02 vaccine represents a promising advancement in the fight against SARS-CoV-2, showcasing the potential of multi-antigen vaccines to provide protection that extends beyond the limitations of first-generation, S-only vaccines. Through its incorporation of the S, M, and E proteins, GEO-CM02 achieves a balanced immune response that combines both humoral and cellular immunity. This is critical as SARS-CoV-2 continues to evolve, making single-antigen vaccines less effective over time. GEO-CM02’s performance in preclinical studies, particularly its ability to protect against lethal SARS-CoV-2 infection, emphasizes the value of targeting multiple antigens to stimulate a broad and durable immune response.

## Data Availability

Data are contained within the article and Supplementary Materials.

## Supplementary Materials

The following supporting information can be downloaded at https://www.mdpi.com/article/10.3390/vaccines13040411/s1, Figure S1: In vivo vaccination and infection schematic; Figure S2: Construction and characterization of GEO-CM02 SARS-CoV-2 vaccine; Figure S3: Humoral response after GEO-CM02 vaccination in hACE2 mice; Figure S4: Neutralization antibodies levels after only one dose of GEO-CM02 in hACE2 mice; Figure S5: FCM Gate diagram; Figure S6: Memory phenotype assessment after GEO-CM02 vaccination in hACE2 mice.
