# Supplementary material for: Multi-Antigen Viral-Vectored Vaccine Protects Against SARS-CoV-2 and Variants in a Lethal hACE2 Transgenic Mouse Model
Source: Vaccines (Basel). 2025 Apr 15;13(4):411. doi: 10.3390/vaccines13040411 (PMC12031414; doi:10.3390/vaccines13040411)
Supplement: Supplementary file 1 [file vaccines-13-00411-s001.zip › vaccines-3478176-supplementary.pdf]

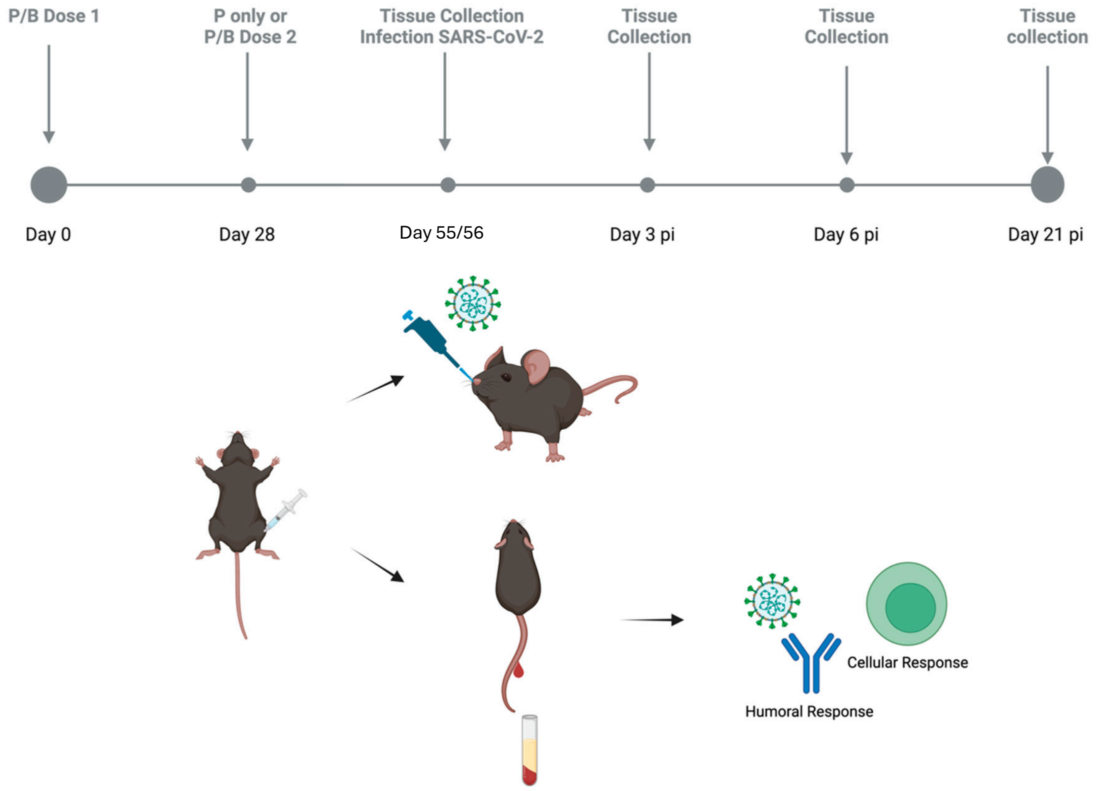

Supplementary Figure S1. In vivo vaccination and infection schematic. Mice were vaccinated with either a single dose (prime) or a prime followed by a booster dose (prime–boost), administered 28 days apart. Some mice were euthanized on day 28 (prime group) and day 55 (prime–boost group) to collect samples for assessing humoral and cellular immune responses. The remaining mice were infected intranasally with either PBS (mock) or 10^5 PFUs of SARS-CoV-2 at 28 days post-prime or 56 days post-prime–boost vaccination. These mice were monitored daily for 3 weeks. A subset of animals was sacrificed on days 3 and 6 post-infection for tissue collection and for analysis of virological and pathological outcomes.

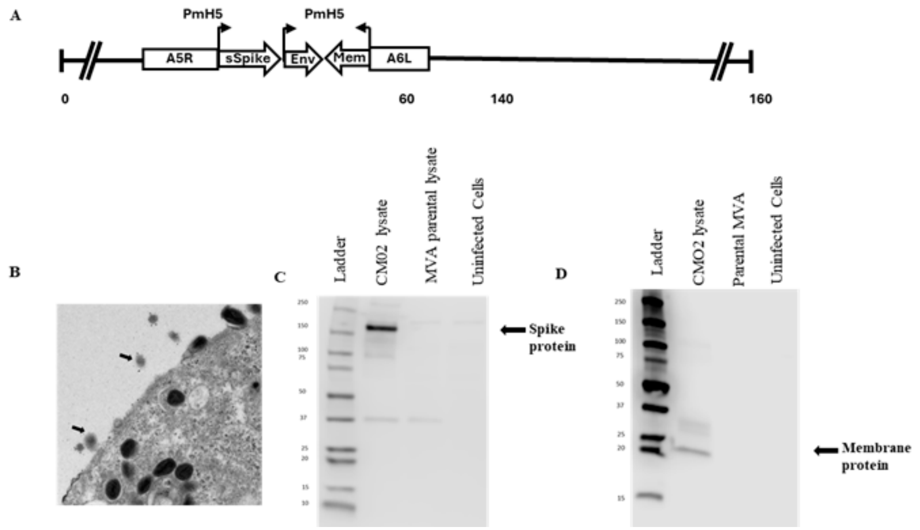

Supplementary Figure S2. Construction and characterization of GEO-CM02 SARS-CoV-2 vaccine. (**A**) Vector construction map. SARS-CoV-2 genes were inserted into one site between two essential MVA genes, A5R and A6L, under the direction of MVA promoter-modified H5 (PmH5). Positions are given in kilobase base pairs in the MVA genome. (**B**) Electron microscopic analysis demonstrating VLP formation indicated by arrows. Thin-layer cell sections infected with GEO-CM0 2 Immunogold labeling carried out using anti-Spike primary antibody (Sino, T62) and immunogold secondary. The scale bars in the bottom left of the image are 200nm. (**C**,**D**) Western blot analysis of GEO-CM02-infected DF-1 cell extracts probed for (**C**) spike protein and (**D**) membrane protein.

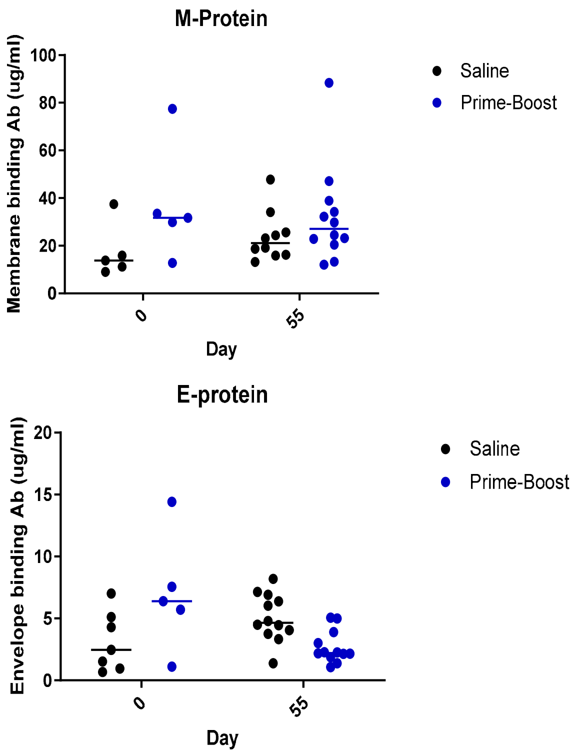

Supplementary Figure S3. Humoral response after GEO-CM02 vaccination in hACE2 mice. IgG binding antibody titers. Binding Ab titers against SARS-CoV-2 B.1-specific M and E proteins were measured in serum samples of vaccine and saline groups at days 0 and 55 after vaccination using ELISA. Data are presented as mean values ± SEM. Each point represents an individual mouse.

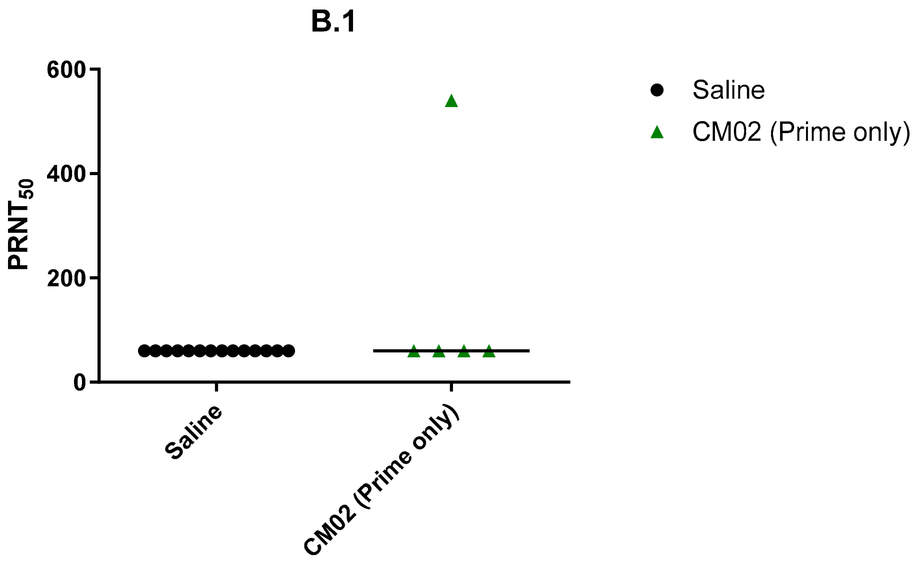

Supplementary Figure S4. Neutralization antibodies levels after only one dose of GEO-CM02 in hACE2 mice. NAb titers in vaccine and saline serum collected at day 28 after vaccination were measured against B.1 live virus by PRNT assay. Titers are expressed as PRNT50. Data are presented as mean values ± SEM. Each point represents an individual mouse.

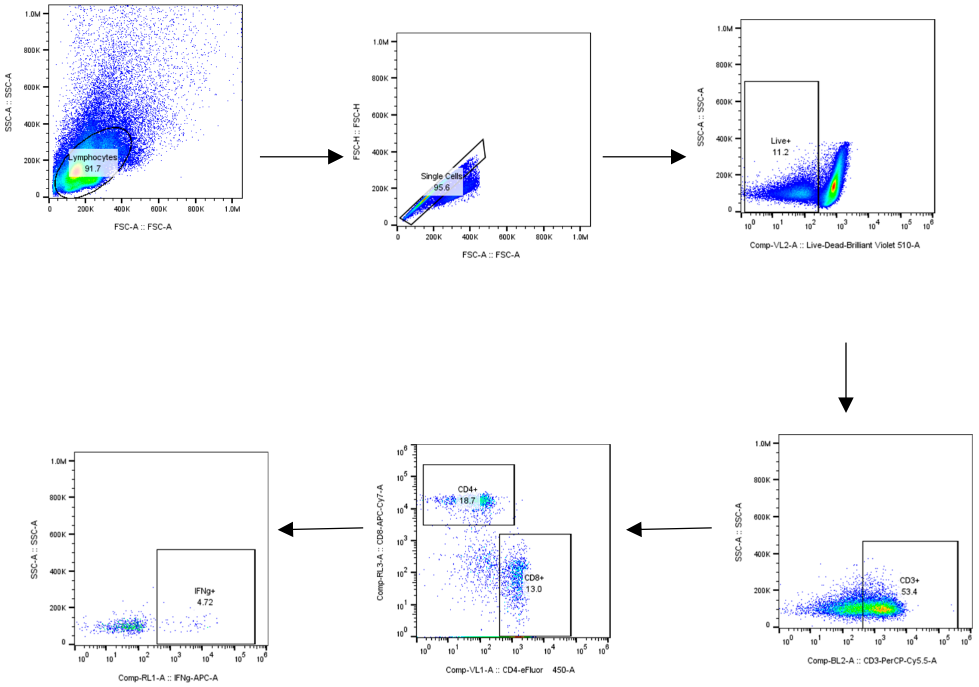

Supplementary Figure S5. FCM Gate diagram. Splenocytes were isolated from spleens collected 4 weeks following saline or GEO-CM02 prime or prime–boost vaccination (day 55) of hACE2 mice. Cells were stimulated ex vivo with B.1-specific S or M peptide pools, fixed, stained for live/dead and cell surface markers (CD3, CD4, and CD8) and intracellular cytokines (IFNγ, IL-2, and IL-4), and analyzed by flow cytometry.

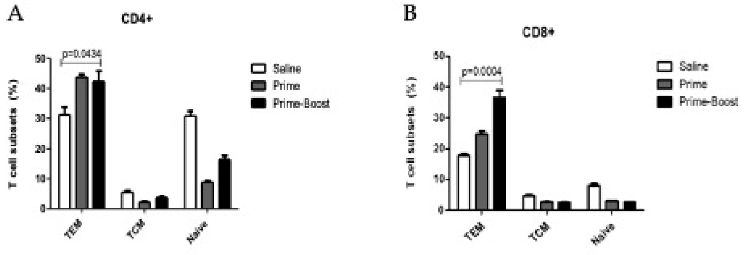

Supplementary Figure S6. Memory phenotype assessment after GEO-CM02 vaccination in hACE2 mice. Splenocytes were isolated from spleens collected 4 weeks following saline or GEO-CM02 prime or prime–boost vaccination (day 55) of hACE2 mice. Cells were stained with CD44 and CD62L and gated for central memory (Tcm), effector memory (Tem), and naïve populations.
